# The Metalloprotease ADAM12 Regulates the Effector Function of Human Th17 Cells

**DOI:** 10.1371/journal.pone.0081146

**Published:** 2013-11-21

**Authors:** Angela X. Zhou, Aimee El Hed, Frances Mercer, Lina Kozhaya, Derya Unutmaz

**Affiliations:** 1 Department of Microbiology, New York University School of Medicine, New York, New York, United States of America; 2 Department of Pathology, New York University School of Medicine, New York, New York, United States of America; 3 Department of Medicine, New York University School of Medicine, New York, New York, United States of America; Emory University School of Medicine, United States of America

## Abstract

A key modulator of immune homeostasis, TGFβ has an important role in the differentiation of regulatory T cells (Tregs) and IL-17-secreting T cells (Th17). How TGFβ regulates these functionally opposing T cell subsets is not well understood. We determined that an ADAM family metalloprotease called ADAM12 is specifically and highly expressed in both Tregs and CCR6+ Th17 cells. ADAM12 is induced *in vitro* upon differentiation of naïve T cells to Th17 cells or IL-17-secreting Tregs. Remarkably, silencing ADAM12 expression in CCR6+ memory T cells enhances the production of Th17 cytokines, similar to suppressing TGFβ signaling. Further, ADAM12 knockdown in naïve human T cells polarized towards Th17/Treg cells, or ectopically expressing RORC, greatly enhances IL-17-secreting cell differentiation, more potently then inhibiting TGFβ signals. Together, our findings reveal a novel regulatory role for ADAM12 in Th17 cell differentiation or function and may have implications in regulating their aberrant responses during immune pathologies.

## Introduction

Th17 cells are characterized by their secretion of IL-17, an inflammation-inducing cytokine that is implicated in the pathogenesis of several autoimmune processes, including asthma, systemic lupus erythematosus, colitis and allograft rejection [1]. Th17 cell differentiation requires the combination of TGFβ and pro-inflammatory cytokines including IL-6, IL-1β and IL-23 [2]. TGFβ is also crucial for generating induced regulatory T cells, a portion of which secrete IL-17, and have anti-inflammatory functions in controlling excessive immune response [3-6]. How TGFβ signals regulate programming of these functionally distinct IL-17-secreting T cell subsets are not well understood.

TGFβ is a pleiotropic cytokine involved in development, fibrosis, wound healing, and immune regulation [7,8]. Part of the regulation of TGFβ occurs through modulation of its signaling. After secretion and processing, mature TGFβ is first recognized by TGFβRII, which then recruits and phosphorylates TGFβRI [8,9]. The receptor-ligand complex is typically endocytosed into early endosomes where it initiates the downstream signaling cascade through R-Smads [10]. A recent study suggested that a member of the ADAM (a disintegrin and metalloprotease) family of metalloproteases, ADAM12, could interact with TGFβRII [11] and that this interaction could enhance TGFβ signaling through control of TGFβR localization and stability on early endosomes [11,12]. 

23 ADAMs have been identified in humans [13]. All are cell-surface proteins containing multiple domains that exert a variety of effects on cell adhesion and migration [13]. In addition, a number of ADAMs, including ADAM12, contain active metalloprotease domains that can mediate “shedding” of cell surface proteins to activate and regulate their functions [14,15]. ADAM12 is expressed primarily in mesenchymal tissues that form skeletal muscle and bone and may have functions during muscle regeneration after injury [16]. In addition, ADAM12 has been implicated in a number of diseases, including musculoskeletal and neurological disorders and cancer [17]. Interestingly, ADAM12 was found to be significantly upregulated on T cells infiltrating the spinal cords of mice in an EAE model, which is mediated by pathological Th17 cell effector functions [18].

In this study, we determined that ADAM12 is highly and specifically expressed in human IL-17-secreting T cells and most Tregs. Knockdown of ADAM12 in primary human memory T cells significantly enhanced the proportion of cells producing IL-22, IL-17A, IL-17F, and both IL-17A and IFNγ, in both TGFβ-dependent and -independent manners. Silencing ADAM12 in naïve cells also greatly enhanced their differentiation into IL-17-secreting T cells. Our results suggest that ADAM12 is an important regulator of Th17 cell differentiation and effector functions. 

## Materials and Methods

### T cell purification

PBMCs from healthy individuals were prepared using Ficoll-Paque plus (GE Healthcare) from discarded buffy coats obtained anonymously from the New York Blood Center (New York, NY). All donor samples were non-identifiable and did not involve any donor-specific information for data analysis and therefore consent forms were not required. All human material was obtained and processed according to guidelines and approval of NYU School of Medicine Institutional Human Subjects Board. CD4+ T cells were isolated using Dynal CD4 Positive Isolation Kit (Invitrogen) and were >99% pure. CD4+ cells were sorted by flow cytometry (FACSAria; BD Biosciences) on the basis of expression of CD45RO and CD25 for naïve T cells, memory T cells, naïve Tregs and memory Tregs as described previously [19]. Sorted subsets were >99% pure and were kept at 37°C and 5% CO2 in Roswell Park Memorial Institute 1640 medium with 10% fetal calf serum.

### T cell activation and infection

CD4+ T cell subsets were stimulated using plate-bound anti-CD3 antibody and soluble anti-CD28 or monocyte-derived dendritic cells (MDDCs) and anti-CD3 (OKT3), and maintained in IL-2-containing media. Activated cells were infected with lentiviruses as indicated. The empty vector and *RORC*-cDNA containing lentiviruses pseudotyped with VSVG envelope were generated as previously described and express GFP as the marker in place of the *nef* gene [20-23]. The lentivirus-encoding *RORC* gene, RORC-IRES-GFP, was a gift from Dr. Dan Littman (New York University School of Medicine, New York, NY). ADAM12 and control lentiviral shRNAs, which encode puromycin selection marker, were purchased from Sigma. For shRNA transductions, 2ug/ml puromycin (Sigma) was added on day 4 post-activation. For *in vitro* polarizations, Tn or naïve Tregs were cultured in IL-1β (10ng/ml), IL-23 (100ng/ml) and TGFβ (10ng/ml) and maintained in IL-2 for 12 days, as previously described [24]. For cytokine staining, cells were reactivated for 5h with Phorbol 12-myristate 13-acetate (PMA 20 ng/mL; Sigma) and ionomycin (500 ng/mL; Sigma) in the presence of GolgiStop (Brefeldin A; BD Biosciences). In the experiments blocking TGFβ signals, cells were either treated with SJN2511 (Tocris bioscience) or TGFβ neutralizing antibody (clone 1D11; R&D) at the time of activation.

### RNA isolation and quantitative RT-PCR

Purified T cells were flash frozen in liquid nitrogen. Total RNA was isolated using RNeasy isolation kit (Qiagen) according to the manufacturer’s protocol, reverse transcribed into cDNA and quantified using an Applied Biosystems 7300 apparatus (Foster City, CA). Taqman primers and probe mixes were purchased from Applied Biosystems, and their IDs are as follows: Human ADAM12 (Hs01106101_m1), ADAM12S (Hs00222216_m1), RORC (Hs01076112_m1), FOXP3 (Hs00203958_ml), ADAM19 (Hs00224960_ml), ADAM22 (Hs00244640_ml), IL-17A (Hs99999082_ml) and β-Actin (Hs99999903_ml).

### Staining and FACS sorting analysis

Cells were stained with corresponding antibodies, as previously described [21]. For intracellular staining, fixation and permeabilization was performed using FOXP3 staining Kit (eBioscience) in accordance with the manufacturer's instructions. Analyses were performed using LSRII flow cytometer (BD Biosciences, San Jose, CA) and FlowJo software (Tree Star Inc, Ashland, OR). The following anti-human antibodies were used for staining: CD25, CD45RO, interferon (IFN)-γ, IL-17A, IL-17F, IL-22 (Biolegend) and CCR6 (BD Biosciences).

### Ex vivo isolation of Th17 cells

Technique for isolation of Th17 cells from total CD4+ T cells was adapted from Streeck et al. [25]. Briefly, T cells were activated for 3.5h using PMA and ionomycin as described above. Cells were washed with ice-cold PBS containing 2% fetal bovine serum and resuspended in PBS and 2% FBS. The IL-17 capture complex containing anti-CD45-biotin combined with anti-IL-17-biotin was prepared and vortexed thoroughly before mixing in free avidin solution to link the two antibodies. The complex was then incubated for 10min at room temperature before adding to cells. After 15 minutes on ice, RPMI was added and cells were incubated at 37C with 5% CO_2_ for 1.5h. Cells were washed again and surface stained using IL-17-PE antibody and sorted on FACS ARIA for IL17+ and IL-17- cells.

### Statistical Analysis

All statistical analyses were performed with GraphPad Prism Software (GraphPad Inc., La Jolla, CA). The significance was determined using One-Sample Student’s t-test.

## Results

### Expression of ADAM12 is enriched in IL-17+ cells

**Figure 1:**
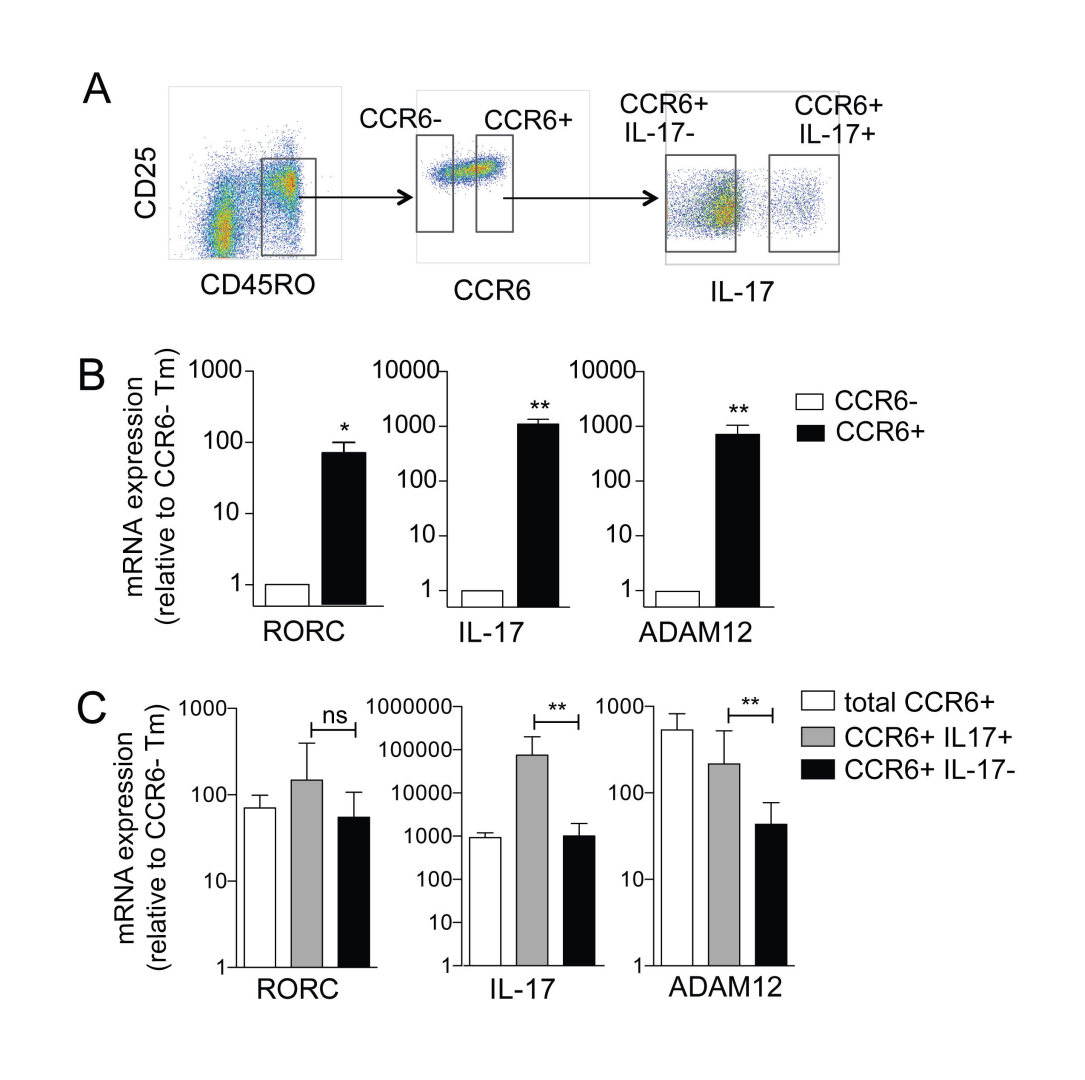
ADAM12 expression in Th17 cells. (**A**) CD4+ T cells isolated from human PBMCs were stained for CCR6, CD25 and CD45RO expression and sorted into CCR6+ and CCR6- Tm (CD25- CD45RO+) cells. Representative FACS plots showing gating for isolation of CCR6+ and CCR6- Tm cells. Cells were subsequently activated by PMA and ionomycin and separated based on secretion of IL-17 as described in materials and methods. (**B**) CCR6+ and CCR6- cells were activated by plate-bound anti-CD3 and soluble anti-CD28 antibody for 16 hours and relative levels of RORC, IL-17 and ADAM12 were determined by qRT-PCR. (**C**) Expression of RORC, IL-17 and ADAM12 in *ex* *vivo* isolated Th17 subsets was determined by qRT-PCR relative to expression on CCR6- Tm cells. * p<0.05, ** p<0.01.

During a microarray analysis of differential gene expression in human T cell subsets [19,26], we observed that a member of the ADAM family of metalloproteases, ADAM12 was specifically expressed in CCR6+ memory T cells (Tm), in a discovery based microarray analysis. We confirmed this preferential and high expression of ADAM12 mRNA in highly purified CCR6+ and CCR6- Tm cells (Figure 1A) through qRT-PCR (Figure 1B). ADAM12 expression in CCR6+ Tm subsets correlated with expression of the Th17 cell signature genes, *RORC* and IL-17 (Figure 1B). 

ADAM12 is also highly expressed on infiltrating T cells in the CNS of an EAE mouse model, which is dependent on dysregulated Th17 cell functions [18]. Based on our finding of preferential expression of ADAM12 in CCR6+ T cells, which contain all Th17 cells, we hypothesized that ADAM12 may be expressed at higher levels in IL-17-secreting Th17 cells. To test this, we adapted a method [25] to isolate IL-17 secreting cells *ex vivo* from CD4+CD45RO+CCR6+ T cells (Figure 1A). Indeed, we found that ADAM12 expression was much higher in IL-17-secreting T cells, and also correlated with *RORC* and IL-17 mRNA expression (Figure 1C).

In order to verify that the preferential expression in CCR6+ Tm cells was unique to ADAM12, we also determined the relative expression of two other ADAM genes: ADAM19 and ADAM22. Like ADAM12, ADAM19 was shown to be upregulated specifically on T cells in the spinal cords of EAE mice, although to a much lower extent [18]. In contrast to ADAM12, we did not observe specific expression of ADAM19 and ADAM22 within CD4+ CCR6+ Tm cell subsets (Figure S1A).

### Silencing ADAM12 expression in memory T cells enhances Th17 cytokine secretion

To examine the role of ADAM12 in CCR6+ Tm cells, we transduced purified CCR6+ cells with lentiviruses encoding ADAM12 shRNA. Using qRT-PCR, we confirmed that the shRNAs silenced gene expression of ADAM12 by 50-80% (Figure S2A). We then reactivated these shRNA-expressing cells through the TCR and assessed for changes in cytokine and transcription factor expression (Figure S2B). 

**Figure 2:**
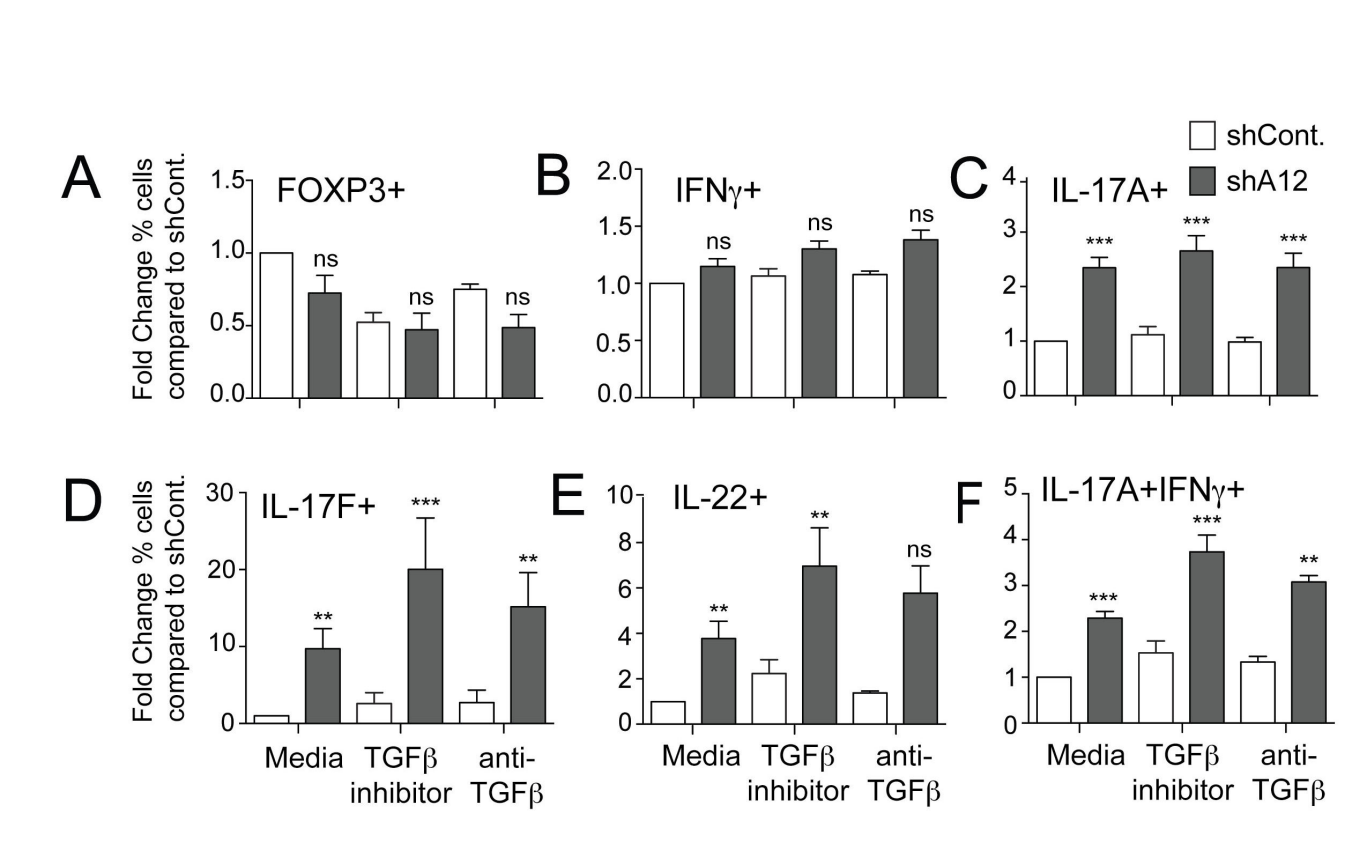
Effect of ADAM12 knockdown on Th17 cytokine production by CCR6+ Tm cells. (**A**) After transduction with control (shCont.) or ADAM12 (shA12) shRNA, CCR6+ Tm cells were activated in media alone, with a TGFβ signaling inhibitor or with TGFβ neutralizing antibodies. Graphs show fold change in percentage of cells that were FOXP3+, (**B**) IFNγ+, (**C**) IL-17A+, (**D**) IL-17F+, (**E**) IL-22+ or (**F**) IL-17A+ IFNγ+ on day 4 post-activation compared to shCont.-tranduced cells in media. Statistical significance ** p<0.01, *** p<0.001.

Based on previous reports describing the potential role of ADAM12 in enhancing TGFβ signaling [11], we hypothesized that the knockdown of ADAM12 would mimic the actions of inhibiting TGFβ signals. We found that CCR6+ Tm cells transduced with ADAM12 shRNA did not impact IFNγ secretion or FOXP3 expression (Figure 2A and B). Strikingly, however, ADAM12 knockdown caused significant increase in the numbers of cells secreting Th17 cytokines, IL-17A, IL-17F, and IL-22 (Figure 2C-E). In addition, populations of cells producing both IL-17A and IFNγ were greatly increased upon silencing ADAM12 expression (Figure 2F). Blocking TGFβ signals with an inhibitor or neutralizing antibody also increased Th17 cytokine production, in line with previous reports that high concentrations of TGFβ is inhibitory on IL-17-secreting cells [27,28], but to a lower extent compared to ADAM12 knockdown (Figure 2C-F). We also observed that blocking TGFβ signals on cells where ADAM12 was silenced could further augment the secretion of Th17 cytokines (Figure 2C-F). Together, these findings suggest that reducing ADAM12 levels is either more potent than blocking TGFβ signals via inhibitors or neutralizing antibodies, or that ADAM12 can also function independent of TGFβ in regulating Th17 cytokines. 

### ADAM12 expression in RORγt-overexpressing cells

**Figure 3:**
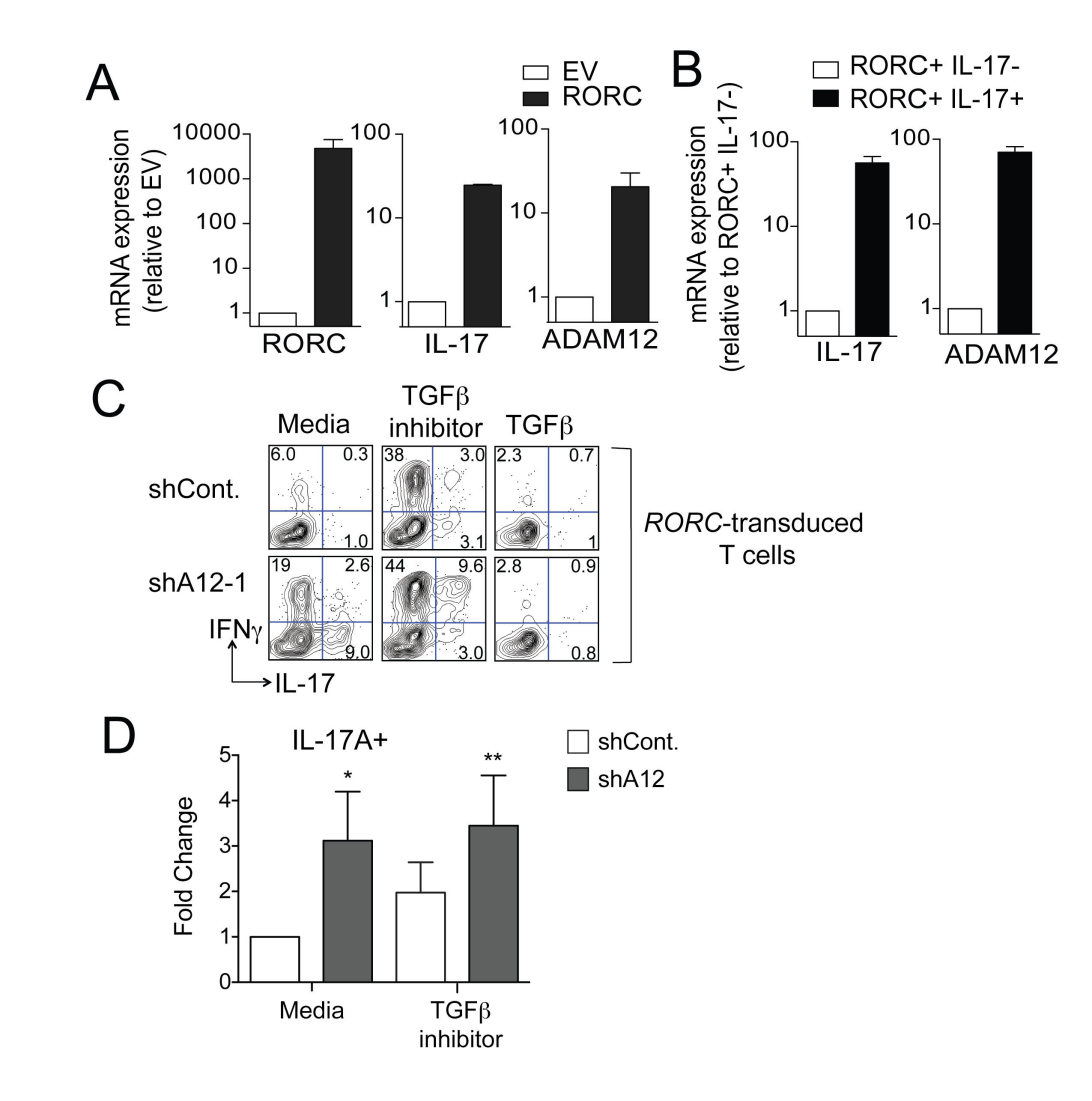
Regulation of Th17 cytokines by ADAM12 expression induced in RORC-transduced Tn cells **(A)** Expression of RORC, IL-17 and ADAM12 was determined in Tn cells transduced with EV or RORC lentiviruses by qRT-PCR. (**B**) IL-17 expressing Tn cells transduced with RORC lentivirus were sorted as described in materials and methods and expression of IL-17 and ADAM12 was determined by qRT-PCR. (**C**, **D**) Tn cells were transduced with both RORC-lentivirus and ADAM12 (shA12-1) or control shRNA (shCont.). After selection, cells were reactivated through the TCR and assessed for expression of IFNγ and IL-17. (C) Representative FACS plots of intracellular cytokine-expression. (D) Percent comparison of IL-17+ cells transduced with shCont or shA12. Statistical significance: * p<0.05, ** p<0.01.

RORγt is a master transcription factor regulator of Th17 cells [29]. Because ADAM12 expression was Th17 cell-specific and correlated with *RORC* expression on Tm cells, we asked whether RORγt was involved in inducing ADAM12 expression. To test this, we transduced Tn cells with an *RORC*-lentivirus, expanded cells in IL-2-and performed qRT-PCR for ADAM12 mRNA expression. We found that *RORC*-transduced cells had also upregulated both IL-17 and ADAM12 expression (Figure 3A). Further, upon sorting IL-17-secreting *RORC*-transduced cells, we found that ADAM12 was also preferentially upregulated in the IL-17+ cells (Figure 3B), similar to our ex vivo observations (Figure 1B and C). To determine the role of ADAM12 on IL-17 expression in these cells, we silenced ADAM12 expression in *RORC*-transduced cells using ADAM12 shRNA, as described above. Similar to CCR6+ Tm cells, the knockdown of ADAM12 in *RORC*-transduced cells resulted in increased expression of IL-17 (Figure 3C and D). Inhibiting TGFβ signals with a TGFβ signaling inhibitor also increased Th17 cytokine production in some donors, whereas treating with exogenous TGFβ inhibited cytokine production completely (Figure 3C). 

### Regulatory T cells also express ADAM12

**Figure 4:**
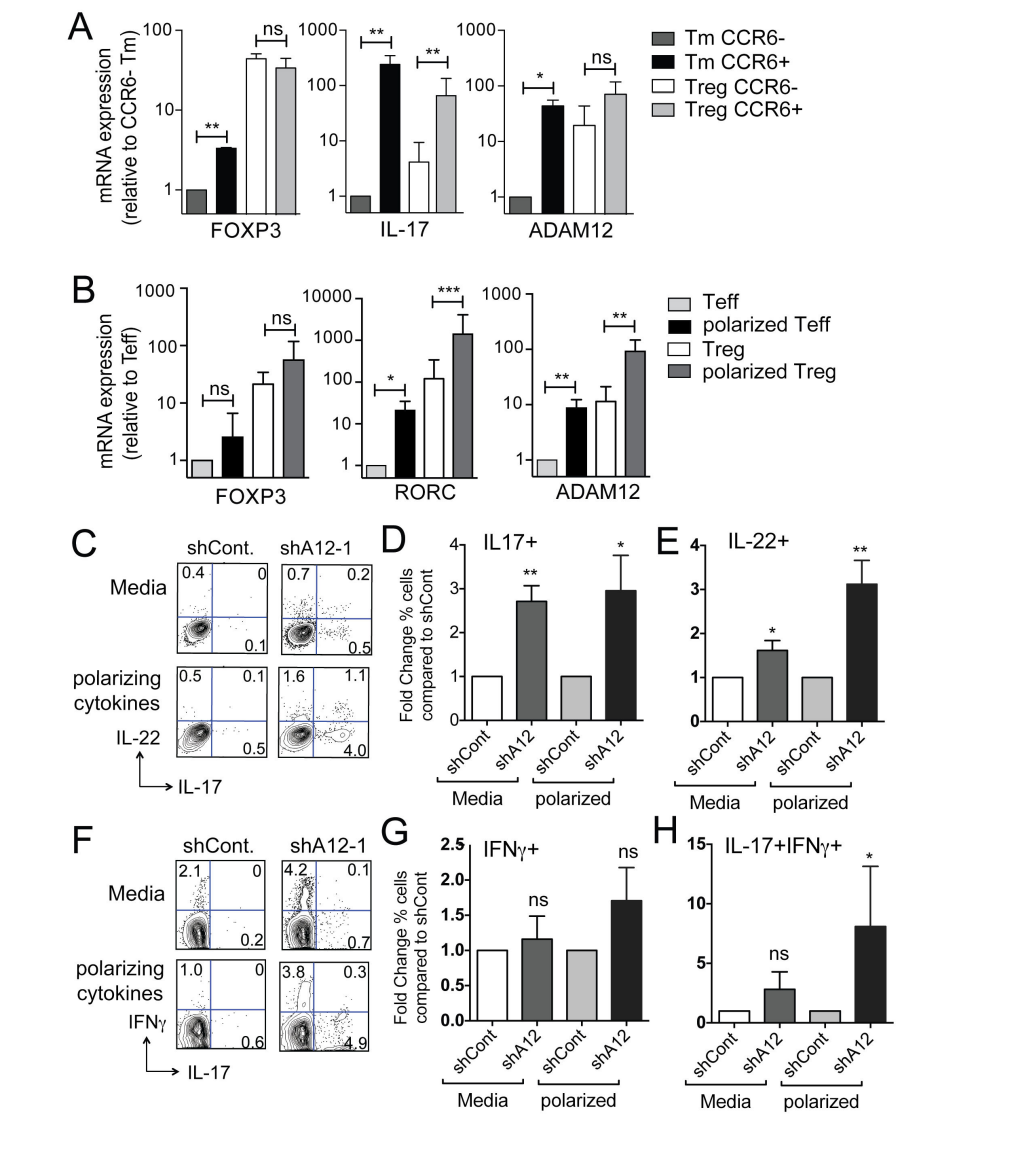
Expression and effects of ADAM12 on polarized Tregs. **(A)** Tm cells and Tregs (CD45RO+ CD25+) were sorted based on surface CCR6 and analyzed for expression of FOXP3, IL-17 and ADAM12 by qRT-PCR. **(B)** Teff and Tregs were activated with or without Th17 polarizing cytokines for 12 days before analyzing for expression of FOXP3, RORC, and ADAM12 by qRT-PCR. **(C-H)** Percent of IL-22, IL-17 and IFNγ production in Tregs activated in IL-2 media or Th17 polarizing cytokines after transduction with either a control (shCont.) or ADAM12 shRNA (shA12-1) was determined by flow cytometry. (C and F) Representative FACS plots of cytokine expression. Percent comparison of : (D) IL-17+ cells (E) IL-22+ cells (G) IFNγ+ cells (H) IL17+IFNγ+. Statistical significance: * p<0.05, ** p<0.01, *** p<0.001.

Because Tregs also require TGFβ for their differentiation [7], we next determined if ADAM12 was expressed in Tregs. We found that, ADAM12 was enriched in all Tregs regardless of CCR6 expression (Figure 4A). However, as expected, IL-17 expression was preferentially expressed in CCR6+ FOXP3+ Tregs (Figure 4A). A recent study demonstrated that IL-17-secreting cells could be expanded from naïve Tregs using a cytokine cocktail of IL-2, IL-23, IL-1β and TGFβ [24]. To determine if ADAM12 was induced during the course of Th17 polarization, we cultured naïve T cells and Tregs in these Th17 polarizing cytokines for 12 days and then extracted RNA for qRT-PCR. We found that ADAM12 expression was upregulated 10-fold in Th17-polarized cells compared to non-polarized cells in both Tregs and Teff cells (Figure 4B). Since silencing ADAM12 enhanced Th17 cytokine secretion in Tm and *RORC*-transduced cells, we asked whether ADAM12 also regulated Th17 cell differentiation from naïve T cells to Th17 cells. To test this, we transduced naïve Tregs with control or ADAM12 shRNA after activation through the TCR, and cultured cells either in IL-2 media or in the polarizing cytokine cocktail. Reducing ADAM12 gene expression, in the presence of polarizing cytokines, further augmented generation of T cells secreting Th17-cytokines (Figure 4C and D), most of which also expressed FOXP3 (data not shown). Remarkably, ADAM12 knockdown increased levels of IL-17 and IL-22 even in the absence of Th17 polarizing cytokines (Figure 4C-E). Similar to Tm cells, IFNγ levels were not affected (Figure 4F and G), however, cells expressing both IL-17 and IFNγ were significantly increased in polarized naïve Tregs (Figure 4F and H)

## Discussion

In this study, we determined that a surface metalloprotease, ADAM12, is highly expressed in both Th17 cells and Tregs. ADAM12 was induced by the Th17 master transcription factor RORγt and Th17 polarizing cytokines, suggesting it is upregulated as part of Th17 programming. We discovered that silencing ADAM12 expression greatly enhanced the production of Th17 cytokines. 

We observed that CCR6+ Tm cells and both CCR6+ and CCR6- Tregs expressed high levels of ADAM12. The preferential expression of ADAM12 in both Tregs and CCR6+ Tm cells supports proposed similarities in the development of Th17 and Treg lineages [7]. TGFβ, which is important for development of both Th17 and Tregs, has also been reported to induce expression of ADAM12 in hepatic stellate cells, fibroblasts and epithelial cells [30-32]. Part of the TGFβ-mediated ADAM12 induction is through proteasomal degradation of SnoN, which was shown to transcriptionally repress ADAM12 in fibroblasts [32]. TGFβ signals may underlie the high levels of ADAM12 in both populations. It is possible that TGFβ might also upregulate ADAM12 through RORγt, which is induced by TGFβ [29], since overexpression of RORγt was found to upregulate ADAM12 as well (Figure 3).

In response to a knockdown of endogenous ADAM12, CCR6+ Tm cells increased secretion of IL-17A, IL-17F and IL-22, which was similar to blocking TGFβ using either a signaling inhibitor or a neutralizing antibody (Figure 2C-F). This potential link between ADAM12 and TGFβ signaling in regulating Th17 function is consistent with previous reports that ADAM12 interacts with TGFβRII, enhancing TGFβ signaling through increasing receptor endocytosis and decreasing its ubiquitination [11]. It is conceivable that, in T cells, ADAM12 localizes and stabilizes endosomal TGFβRII, leading to amplification of TGFβ signals, as a mechanism to downregulate production of pro-inflammatory cytokines. Since ADAM12 is also upregulated by TGFβ, this would suggest a positive feedback relationship between TGFβ and ADAM12. However, using the TGFβ inhibitors alone did not significantly enhance cytokine secretion in Tm cells, suggesting that ADAM12 may also act through signaling pathways independent of those blocked by TGFβ inhibitors in Tm cells. Future studies dissecting the molecular mechanisms of ADAM12 functions in T cells could provide an important framework for understanding how the Treg-Th17 equilibrium is maintained.

Knockdown of ADAM12 in polarized naive cells either through *RORC* transduction or culture in Th17 polarizing cytokines also enhanced Th17 cytokine secretion. Inhibition of TGFβ signals in these *RORC*-transduced Tn cells, also enhanced their cytokine production, particularly secretion of IFNγ. This finding suggests a phenotypic difference in the sensitivity of naïve versus memory T cells to TGFβ-inhibitor mediated enhancement of cytokine production and might be caused by the suppressive effects of higher levels of TGFβ on T cell differentiation [28]. It has recently been shown that IL-23 potentiates inflammatory function of Th17 cells [33,34]. In the absence of IL-23 signals, RORγt-expressing cells have limited inflammatory capacity, despite upregulation of IL-17 [2]. One mechanism that TGFβ uses to block generation of Th17 cells is by inhibiting expression of IL-23R [35]. In contrast, Tm cells have already completed the differentiation program and may therefore be more resistant to modulatory effects of TGFβ signals. 

Both CCR6+ and CCR6- Tregs express high levels of ADAM12, which is further enriched in the IL-17-secreting Tregs. This is likely due to TGFβ signals, which are involved in differentiation of both natural and induced Tregs [4-6,36]. ADAM12 may be one factor that limits the capacity of Tregs to produce effector cytokines and is therefore necessary in all Treg subsets [37]. Our findings suggest that ADAM12 expression in plays a similar role in regulating IL-17-secretion in a subset of Tregs that can express this cytokine. However, it is also plausible that ADAM12 plays additional role in Treg function given its more broad expression in Tregs.

In summary, our results suggest that ADAM12 functions to repress secretion of Th17 cytokines and thereby act as negative feedback modulator of IL-17 secretion to prevent the potentially destructive nature of inflammatory cytokines on host tissue. It will be important to determine the levels of ADAM12 in T cells in pathologies where Th17 cells are implicated, such as multiple sclerosis patients, psoriasis and other inflammatory disorders. Future approaches to target ADAM12 expression could be an important therapeutic strategy during Th17-associated pathologies. In addition, ADAM12 could act through a novel mechanism to regulate IL-17-secreting Treg and Tm cell differentiation and functions, thus is a potential target to modulate Treg and Th17 cell actions in the context of inflammation and autoimmunity.

## Supporting Information

Figure S1 **Specificity of ADAM12 expression in CCR6+ Tm cells.** Expression of ADAM12, ADAM19 and ADAM22 in CCR6+ and CCR6- Tm cells was determined by qRT-PCR.
(TIF)

Figure S2 **Knockdown of ADAM12 through shRNA expression.** (**A**) Levels of ADAM12 mRNA in CCR6+ Tm cells transduced with shADAM12 (shA12-1 or shA12-2) as a percentage of ADAM12 mRNA from cells transduced with control shRNA (shCont.) was determined by qRT-PCR. (**B**) Purified CCR6+ Tm cells were transduced with shRNA against ADAM12 at the time of activation. After 4 days, puromycin was added to select for cells expressing the lentivirus. Live cells were purified and reactivated by MDDCs and anti-CD3 antibody before assessing cytokine and FOXP3 expression.
(TIF)
