# Supplementary figures and images for: The Metalloprotease ADAM12 Regulates the Effector Function of Human Th17 Cells
Source: PLoS One. 2013 Nov 21;8(11):e81146. doi: 10.1371/journal.pone.0081146 (PMC3867213; doi:10.1371/journal.pone.0081146)

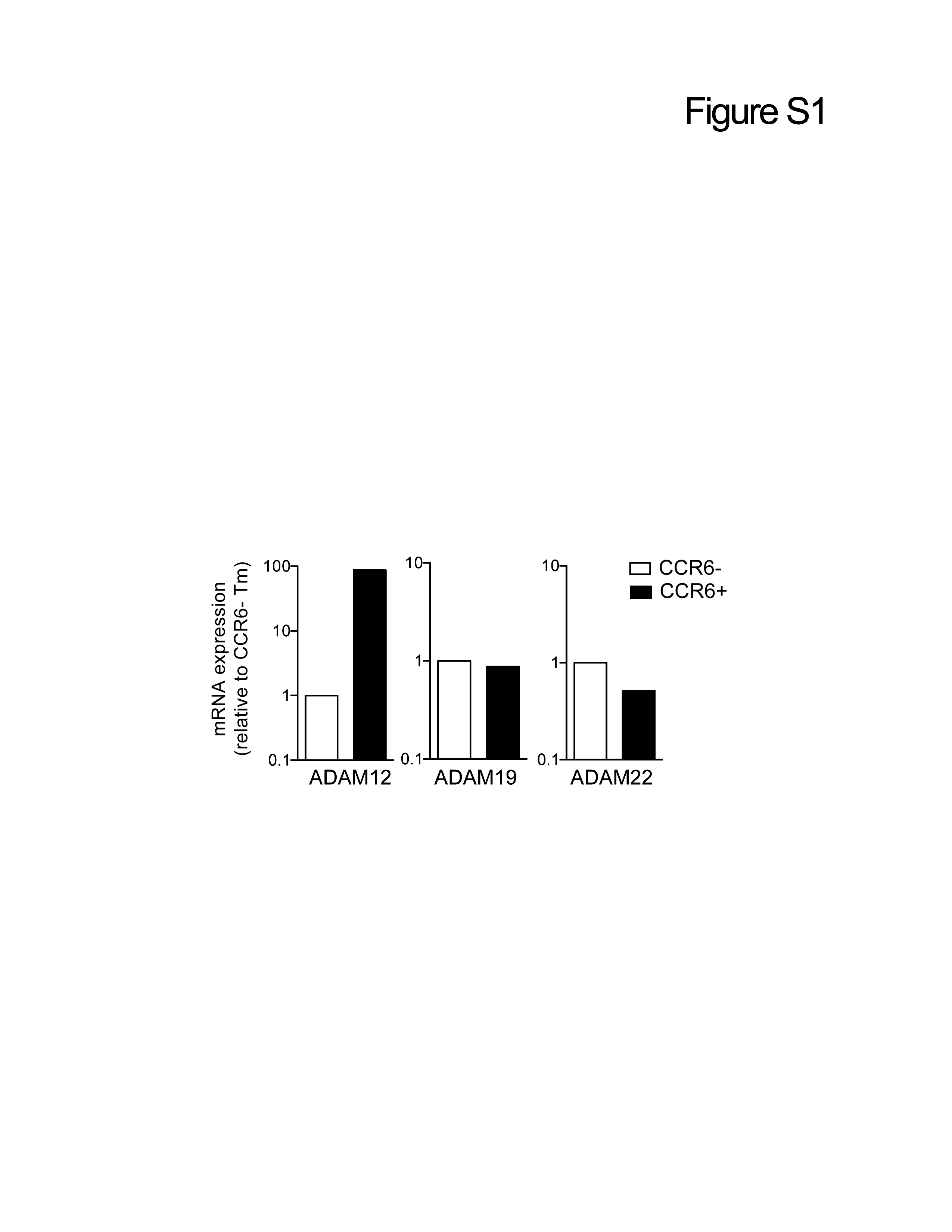

Supplement: Figure S1 — Specificity of ADAM12 expression in CCR6+ Tm cells. Expression of ADAM12, ADAM19 and ADAM22 in CCR6+ and CCR6- Tm cells was determined by qRT-PCR. (TIF) [file pone.0081146.s001.tif]

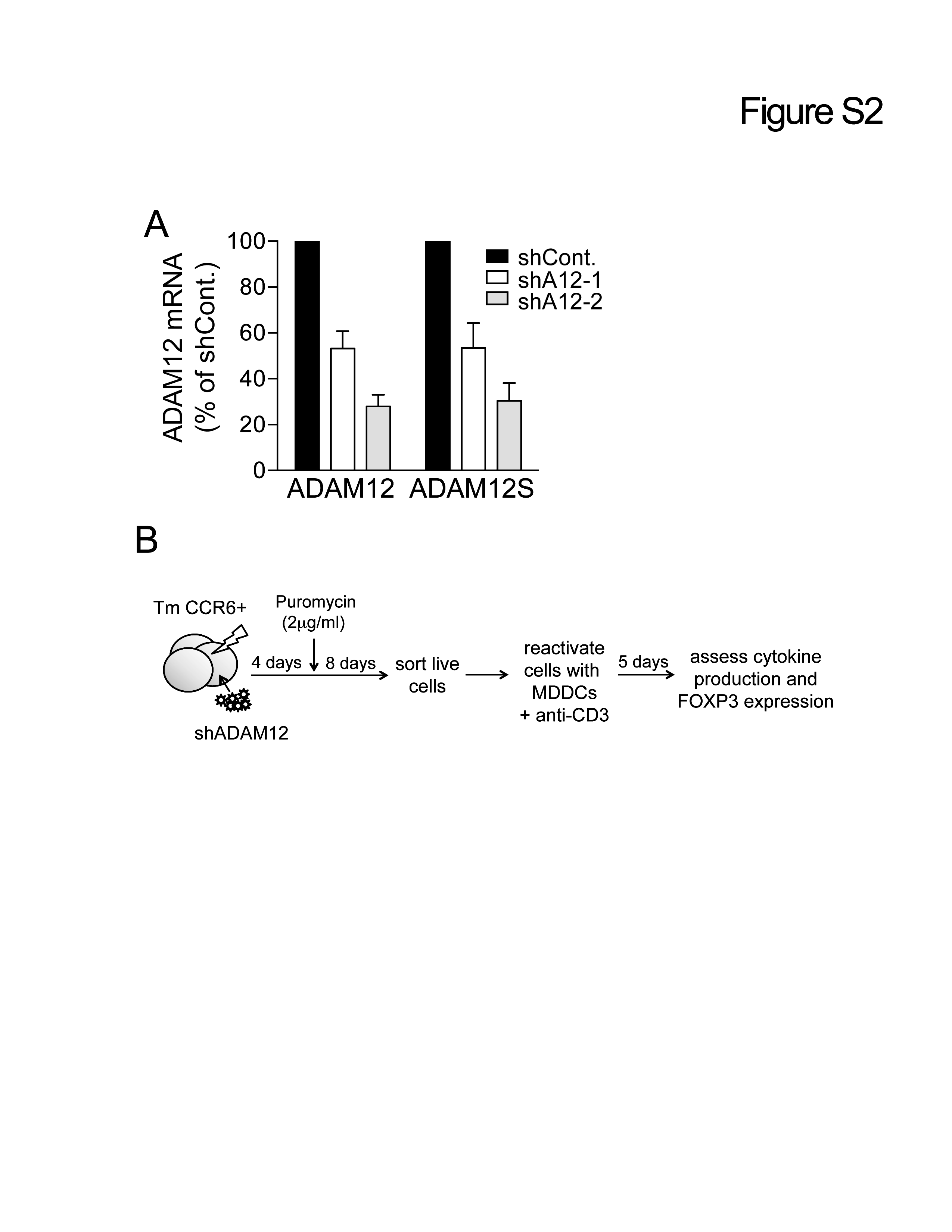

Supplement: Figure S2 — Knockdown of ADAM12 through shRNA expression. (A) Levels of ADAM12 mRNA in CCR6+ Tm cells transduced with shADAM12 (shA12-1 or shA12-2) as a percentage of ADAM12 mRNA from cells transduced with control shRNA (shCont.) was determined by qRT-PCR. (B) Purified CCR6+ Tm cells were transduced with shRNA against ADAM12 at the time of activation. After 4 days, puromycin was added to select for cells expressing the lentivirus. Live cells were purified and reactivated by MDDCs and anti-CD3 antibody before assessing cytokine and FOXP3 expression. (TIF) [file pone.0081146.s002.tif]
